# A Mouse Model of X-Linked Chronic Granulomatous Disease for the Development of CRISPR/Cas9 Gene Therapy

**DOI:** 10.3390/genes15060706

**Published:** 2024-05-28

**Authors:** Seren Sevim-Wunderlich, Tu Dang, Jana Rossius, Frank Schnütgen, Ralf Kühn

**Affiliations:** 1 Max-Delbrück-Center for Molecular Medicine in the Helmholtz Association (MDC), 13125 Berlin, Germany; serensevim1@gmail.com (S.S.-W.); tu.dangngoc@mdc-berlin.de (T.D.); jana.rossius@mdc-berlin.de (J.R.); 2 Department of Medicine, Hematology/Oncology, University Hospital Frankfurt, Goethe University, 60590 Frankfurt am Main, Germany; schnuetgen@em.uni-frankfurt.de; 3 German Cancer Consortium (DKTK), Partner Site Frankfurt/Mainz, and German Cancer Research Center (DKFZ), 69120 Heidelberg, Germany; 4 Frankfurt Cancer Institute, Goethe-University Frankfurt, 60596 Frankfurt am Main, Germany

**Keywords:** X-CGD, CRISPR/Cas9, gene therapy, mouse disease model

## Abstract

Chronic granulomatous disease (CGD) is an inherited immunodeficiency disease mainly caused by mutations in the X-linked *CYBB* gene that abrogate reactive oxygen species (ROS) production in phagocytes and microbial defense. Gene repair using the CRISPR/Cas9 system in hematopoietic stem and progenitor cells (HSPCs) is a promising technology for therapy for CGD. To support the establishment of efficient and safe gene therapies for CGD, we generated a mouse model harboring a patient-derived mutation in the *CYBB* gene. Our CybbC517del mouse line shows the hallmarks of CGD and provides a source for Cybb-deficient HSPCs that can be used to evaluate gene-therapy approaches in vitro and in vivo. In a setup using Cas9 RNPs and an AAV repair vector in HSPCs, we show that the mutation can be repaired in 19% of treated cells and that treatment restores ROS production by macrophages. In conclusion, our CybbC517del mouse line provides a new platform for refining and evaluating novel gene therapies and studying X-CGD pathophysiology.

## 1. Introduction

Chronic granulomatous disease (CGD) is a rare inherited immunodeficiency with an estimated incidence of 1 in about 250,000 individuals worldwide [1,2]. Clinically, CGD is characterized by severe recurrent bacterial and fungal infections, which remain the most significant cause of early mortality. CGD is caused by a failure of phagocytic leukocytes (macrophages, neutrophils) to generate reactive oxygen species, which are needed to kill phagocytized microorganisms [3]. The biochemical defect in CGD lies in NADPH oxidase, the enzyme complex responsible for producing antimicrobial oxygen species in leukocytes [3,4]. NADPH is a multi-component enzyme located in the membranes of leukocytes. The four components of the NADPH oxidase implicated in CGD are gp91*^phox^*, p22*^phox^*, p47*^phox^*, and p67*^phox^* [4]. Two of these proteins, gp91*^phox^* and p22*^phox^*, form the membrane-associated flavocytochrome b558, the actual enzymatic center of the NADPH oxidase [3,4]. Defects in any of the four subunits can manifest as CGD. The most common form of CGD is X-linked recessive, resulting from a mutation of the *CYBB* gene encoding the protein gp91*^phox^* (also known as NOX2). *CYBB* (OMIM *300481) is located on the X chromosome (at Xp21.1) and accounts for the majority (~70%) of CGD cases. According to data from two major databases, there are over 200 mutations in the *CYBB* gene that are linked to X-CGD. The Human Gene Mutation Database (HGMD) at the Institute of Medical Genetics in Cardiff, Wales, lists 282 mutations [5]. Similarly, the Immunodeficiency Database (IDbases) at the Institute of Medical Technology–Bioinformatics in Tampere, Finland, reports 244 mutations [6]. The HGMD database indicates that 58% of these mutations are single-nucleotide substitutions (missense or nonsense, including those affecting splicing), 26% are small deletions, insertions, or indels, and 14% are large deletions or insertions. The remaining 30% of CGD cases follow an autosomal recessive inheritance pattern and result from mutations in p22*^phox^* (*CYBA*) or the cytoplasmic subunits, p47*^phox^* (*NCF1*) and p67*^phox^* (*NCF2*) [7,8]. Mutations in those genes usually lead to partial or complete loss of the respective protein component due to mRNA and/or protein instability [7]. Currently, lifelong antimicrobial prophylaxis is the only treatment for CGD patients [9]. Although a long-term cure for CGD does not exist at this time, stem-cell transplantation and gene therapy are promising curative treatment options. Allogenic transplantation of normal bone marrow has proved beneficial for treating X-CGD. However, this approach is complicated by the risk of transplantation-related morbidity and mortality and limited matched-donor availability [10]. Gene therapy using autologous hematopoietic stem and progenitor cells (HSPCs) requires efficient and safe correction of the patient-derived mutation.

The CRISPR/Cas9 system has emerged as a powerful tool for precise gene editing in mammalian cells. Cas9 nucleases are guided by single-guide RNAs (sgRNAs) to a specific genomic locus, where the nucleases introduce double-stranded breaks (DSB) into the DNA. Gene editing at the target region is achieved by two alternative DSB-repair pathways, either by non-homologous end joining (NHEJ), which leads to randomly sized small deletions or insertions (indels), or by homology-directed repair (HDR), which enables precise sequence modifications that are copied from a donor template molecule. Recently, Ravin et al. [10] have utilized CRISPR/Cas9 to repair a mutation in the *CYBB* gene of CD34+ HSPCs from X-CGD patients. Their HDR-mediated gene-repair approach resulted in functional restoration of NADPH oxidase at clinically relevant levels [10]. Although these promising data have shown the feasibility of the CRISPR method for precise gene correction in X-CGD, the inherent limitations of working with human HSPCs remain. One major challenge is the insufficient number of autologous HSPCs from X-CGD patients. The lack of patient material complicates the in vivo evaluation of the gene-repair strategy because a significant number of corrected cells are needed for transplantation in immunodeficient recipient mice. As an alternative to human HSPCs, mouse models carrying patient-derived mutations could serve as an unlimited source of uniform cellular material and genotype and overcome the limitation associated with using human donors for the establishment of gene therapies. HSPCs are suitable for ex vivo gene repair with subsequent engraftment of corrected cells in recipient mice. In addition, mouse models with the patient-derived mutations allow the exploration of new techniques for in vivo gene repair of stem cells in bone marrow. and can be studied upon challenge with relevant pathogens.

Here, we report the generation of the first mouse model (referred to as CybbC517del mice) carrying a patient-derived mutation for X-CGD (c.517delC [11], IV-33 [3]). This mutation is identical in humans and mice, ensuring that our mouse model accurately represents the human mutation involved in X-CGD. We applied CRISPR/Cas9 to delete a single nucleotide (C517) in exon 6 of the *Cybb* gene. This deletion leads to a reading frameshift beyond codon 173 and Cybb deficiency. gp91*^phox^* expression and NADPH oxidase activity are absent in neutrophils and macrophages from homozygous Cybb-deficient (CybbC517del) mice. We also describe an HDR strategy for genetic correction of this mutation (c.517delC) using CRISPR/Cas9 RNPs and AAV6 viral vectors carrying the repair template. Gene editing in mouse hematopoietic stem and progenitor cells (HSPCs) isolated from CybbC517del mice with constitutive Cas9 expression resulted in a correction rate at clinically relevant levels. Mature macrophages differentiated from repaired CybbC517del HSPCs recover gp91*^phox^* expression and exhibit significant NADPH oxidase activity compared to untreated CybbC517del cells. Furthermore, we confirmed that our HDR strategy did not lead to any detectable off-target effects at the most prominent predicted off-target regions and therefore ensured the safety of our gene-editing approach. Here, we present a valuable disease model for establishing and refining personalized gene-therapy strategies as a potential treatment for X-CGD.

## 2. Materials and Methods

### 2.1. Generation of CybbC517del Mice

CybbC517del mutant mice were generated by CRISPR/Cas9-mediated modification in zygotes. Two sgRNAs flanking codon 173 in exon 6 of the *CYBB* gene were designed using the CRISPOR tool and tested for in vitro cleavage efficiency using a PCR product spanning the target sequence. sgCybb#1 and sgCybb#2 exhibiting high cleavage efficiency were used for further experiments. Zygotes obtained from superovulated C57BL/6N females (Charles River, Sulzbach, Germany) were electroporated as described [12] with sgRNA/Cas9 RNPs complexes and a 125 nt single-stranded DNA oligonucleotide (IDT) that included the C517 deletion as a donor template. The electroporated zygotes were transferred into pseudo-pregnant NMRI females to obtain mutant founder mice. Animals were handled according to institutional guidelines under experimental procedures approved by the Landesamt für Gesundheit and Soziales (Berlin, Germany).

### 2.2. Genotyping of Animals

To genotype ROSA26-Cas9-CybbC517del mice for the specific point mutation in exon 6 of the *CYBB* gene, a 314-bp region was amplified using Cybb-for1 and Cybb-rev1 primers (Table 1) using DreamTaq Polymerase (Thermo Scientific, Darmstadt, Germany). The PCR program included an initial denaturation at 95 °C for 3 min, followed by 35 cycles of denaturation, annealing (62 °C), and extension, with a final extension at 72 °C for 5 min. PCR products were verified on a 2% agarose gel before purification using the GeneJET PCR Purification Kit (Thermo Scientific). Purified PCR products were sent for Sanger sequencing with the Cybb-for1 primer.

Additionally, to confirm constitutive Cas9 expression, genomic DNA was amplified using Cas9-fw and Cas9-rev primers (Table 1), producing a 386 bp product indicative of the Cas9 expression cassette in the Rosa26 locus. This PCR followed a similar thermal-cycling protocol, with annealing at 61 °C. PCR products were analyzed on a 2% agarose gel.

### 2.3. Cybb Repair Template Cloning and rAAV6-Cybb Production

The repair template for the C517 deletion was generated by ligation of a 2000 bp synthetic gBlock DNA fragment (IDT) that included the exon 6 wildtype sequence and two silent nucleotide replacements into an AAV plasmid between the ITR regions of AAV2 (CellBiolabs). Recombinant AAV6-Cybb production with the coat of serotype 6 was performed as previously described [13,14,15]. Briefly, 5 million Hek293T cells were seeded per 150-mm dish in DMEM supplemented with 10% FBS, 1% penicillin/streptomycin, and 25 mM HEPES and grown to 80–90% confluency. The cells were co-transfected with pAAV-Helper (10 µg), pAAV6-Rep/Cap (5 µg), and AAV6-Cybb (5 µg) plasmids using polyethyleneimine (PEI). After four days, the cell pellet and supernatant were harvested and processed separately before they were pooled together. The supernatant was filtered through a 0.22 µm PES membrane and centrifuged at >3000× *g* for 30 min. The cleared supernatant was transferred into a new Falcon tube and incubated in a 5× solution of 40% Polyethylene Glycol 8000 (PEG)/2.5 M NaCl (final concentration 8% PEG/500 mM NaCl) overnight at 4 °C. The next day, the virus was precipitated by centrifugation at >3000× *g* for 30 min. The supernatant was discarded, and the virus precipitate was resuspended in 2 mL PBS/0.001% pluronic F68/200 mM NaCl and kept on ice. The cell pellet was resuspended in 2 mL PBS/0.001% pluronic F68/200 mM NaCl and lysed by three cycles of thaw-freeze on dry ice and 37 °C (10 min per cycle). After centrifugation at >3000× *g* for 15 min, the cleared lysate was combined with the virus resuspension from the supernatant and treated with DNA endonuclease Benzonase (50 units per mL of virus suspension) for 1.5 h at 37 °C. After cleaning by centrifugation, the virus-containing supernatant was prepared for iodixanol gradient ultracentrifugation. In an iodixanol gradient tube (Beckmann, Krefeld, Germany), the layers of gradients were overlayed by a 10 mL syringe with a long 18-gauge needle, as follows: 8 mL of 15% iodixanol, 6 mL of 25% iodixanol, 5 mL of 40% iodixanol, and 5 mL of 60% iodixanol, respectively. The translucent supernatant containing the virus was dripped slowly onto the top layer of the gradient tube using a 20-gauge needle. The tubes were centrifuged at 58,000 rpm for 2 h 10 min at 18 °C in a Beckman Type 70Ti rotor. After ultracentrifugation, the layer from the 40–60% iodixanol interface was extracted carefully via an 18-gauge needle and filtered through a 0.22 µm PES membrane. The virus solution was dialyzed overnight twice with PBS containing 0.001% F68 at 4 °C in dialysis cassettes (Thermo Scientific). Finally, the virus solution was concentrated using Amicon Ultra-15 100-kDa MWCO columns (Millipore, Darmstadt, Germany) until 500 µL^−1^ mL of concentrated virus solution remained. The concentration of the virus was measured by real-time PCR using TaqMan probes (Table 1) specific for the left and right AAV inverted terminal repeats (ITRs).

### 2.4. Isolation and Culture of Mouse Sca-1^+^ Hematopoietic Stem and Progenitor Cells

The isolation of Sca-1^+^ cells was performed as described by Tran et al. [15]. Cells were isolated from the bone marrow of both ROSA26-Cas9-CybbC517del mice and wild-type littermates using the Sca1 enrichment kit according to the manufacturer’s manual (Miltenyi Biotec, Bergisch Gladbach, Germany). After isolation, the Sca-1^+^ cells were resuspended in 2 mL of serum-free HPSC medium (StemSpanTM SFEM II medium supplemented with mouse SCF (50 ng/mL), mouse TPO (50 ng/mL), mouse Flt3-L (50 ng/mL) and human IL-11 (50 ng/mL)) and seeded at a density of 2 × 10^5^ cells/mL in a well of a six-well plate.

### 2.5. sgRNA Electroporation and AAV Transduction

For sgRNA complex formation, crRNA and tracrRNA oligos were mixed in equimolar concentrations (5 µL of 200 µM each) in a sterile PCR tube, incubated at 95 °C for 5 min in a thermal cycler, and cooled to room temperature. Then, 48 h after isolation, Sca-1^+^ cells were collected in a 15 mL falcon tube and the total cell count was determined. The cells were washed once with PBS at room temperature, and 3 × 10^5^ cells were resuspended in 20 µL of AMAXA electroporation buffer. To this suspension, 1.2 µL (120 pmol) of the hybridized cr:tracrRNA complex was added. The final electroporation mixture was then carefully transferred to a well of a 16-well Nucleocuvette strip. Electroporation was conducted using the AMAXA 4D-NucleofectorTM (Lonza), following the program specified for ‘mouse B cells’ [15]. Immediately afterwards, 80 µL of pre-warmed complete HSPCs medium was added to each well. The cells were then transferred to a well in a six-well plate containing 1.5 mL of the same medium. Thirty minutes later, the cells were transduced with AAV6-Cybb at a multiplicity of infection (MOI) of 1 × 10^6^ genome copies per cell. Two days after electroporation, the cells were initiated into macrophage differentiation.

### 2.6. Differentiation of HSPCs into Mature Macrophages

Mouse HSPCs were cultured in serum-free HSPC medium (StemSpanTM SFEM II medium supplemented with mouse SCF (50 ng/mL), mouse TPO (50 ng/mL), mouse Flt3-L (50 ng/mL) and human IL-11 (50 ng/mL)) for four days after isolation. At day four, the medium was replaced with a macrophage-differentiation medium supplemented with GM-CSF (20 ng/mL). The cells were then cultured for an additional 14 days in this complete macrophage medium. Mature macrophages were identified by flow cytometry using APC anti-mouse F4/80 (Rat IgG2a, κ, Clone: BM8).

### 2.7. Off-Target Analysis by T7 Endonuclease I (T7EI) Cleavage Assay

Off-target sites for sgRNA-Cybb were selected using CRISPOR (http://crispor.tefor.net/ (accessed on 14 May 2021)). Wizard Genomic DNA Purification Kit (Promega, Walldorf, Germany) following the manufacturer’s instructions. For the on-target site, PCR was done using PrimeStar GXL (Takara, Mountain View, CA, USA) with Cybb-geno-for and Cybb-geno-rev primers (Table 1) using the following conditions: 98 °C for 2 min, 30 cycles (98 °C for 10 s, 55.1 °C for 10 s, 68 °C for 50 s), and 68 °C for 10 min. For the off-target sites, PCR was done using Q5^®^ High-Fidelity DNA Polymerase (NEB) with site-specific primers (Table 1) using the following conditions: 98 °C for 30 s, 35 cycles (98 °C for 10 s, 56.2 °C (for Actn1 site)/62 °C (for Chd5, Tmem82 and K23Rik sites)/60 °C (for Chchd7, Tceb3 and Qdpr sites) for 30 s, 72 °C for 30 s), and 72 °C for 2 min. PCR amplicons were purified using GeneJET PCR Purification Kit (ThermoFischer Scientific).

The purified PCR products were denatured and annealed in NEBuffer 2 (NEB) in a thermocycler using the following conditions: denaturation at 95 °C for 5 min, re-annealing at 95–85 °C (−2 °C/s) and 85–25 °C (−0.1 °C/s). For the T7EI assay, 1000 ng of the heterocomplex PCR product were digested with T7EI (NEB) according to the manufacturer’s instructions. Cleaved DNA fragments were separated on 2% agarose gels, and the DNA concentration of each band was quantified by the ImageJ version 1.53t software. The percentages of HDR and NEHJ rates was calculated as previously described.

### 2.8. On- and Off-Target Analysis by Amplicon Sequencing

The quantification of on- and off-target editing frequency was performed by Illumina amplicon sequencing via Genewiz (Amplicon EZ; GENEWIZ Germany GmbH, Leipzig, Germany). For this purpose, genomic DNA (gDNA) was extracted from targeted and control cells using the Wizard Genomic DNA Purification Kit (Promega) following the manufacturer’s protocol.

For the on-target analysis, an outer PCR reaction (2.2 kb) was performed using primers (Table 1) located outside the homology arms, while a second PCR reaction was performed using primers amplifying a shorter PCR product of 274 bp. For the first PCR reaction, primers Cybb-geno-for and Cybb-geno-rev (Table 1) were used along with PrimeStar GXL DNA polymerase (Takara) and 200 ng DNA per reaction under the following conditions: initial denaturation at 98 °C for 2 min, followed by 30 cycles (98 °C for 10 s, 55.1 °C for 10 s, 68 °C for 50 s), and final extension at 68 °C for 10 min. The second PCR amplification was conducted with Cybb-Amplicon-fow and Cybb-Amplicon-rev (Table 1), Q5 High Fidelity DNA-Polymerase (NEB), and 20 ng of purified 1^st^ PCR product under the following conditions: initial denaturation at 98 °C for 30 s, 30 cycles (98 °C for 10 s, 61 °C for 30 s, 72 °C for 20 s), and final extension 72 °C for 2 min.

For the off-target sites, PCR was done using Q5^®^ High-Fidelity DNA Polymerase (NEB) with site-specific primers (Table 1) using the following conditions: 98 °C for 30 s, 35 cycles (98 °C for 10 s, 56.2 °C (for Actn1 site)/62 °C (for Chd5, Tmem82 and K23Rik sites)/60 °C (for Chchd7, Tceb3 and Qdpr sites) for 30 s, 72 °C for 30 s), and 72 °C for 2 min. The PCR products were purified on columns using the GeneJET PCR Purification Kit (Thermo Scientific, #K0702). Next, 2 µg of each purified PCR reaction was submitted for deep sequencing. 

A custom amplicon-sequencing analysis pipeline licensed from Bioinformatics Expert UG (Berlin, Germany) was employed for data analysis. This pipeline requires the raw data fastq files for paired reads and the reference sequence provided as a fasta file. The core functionalities of the pipeline were implemented using R (version 3.6.0). The alignment algorithm utilized the “pairwiseAlignment()” function from the R package Biostrings (version 2.52.0). Initially, paired reads were aggregated and cleaned to minimize potential sequencing errors. The process for each read pair included the following steps. First, the R2 read was converted to its reverse complement and the quality score was reversed. Second, the R1 and R2 reads were locally aligned to identify the overlapping region, using high penalty values for gap opening and extension to prevent gaps that could misalign bases during aggregation. Third, the R1 read and the reverse complement of the R2 read (rcR2) were stitched together at the overlap, with the base with the highest quality score selected at positions with conflicts and missing bases filled with “N” at the ends. Fourth, the initial 15 bases from the 5′ and 3′ ends of the reference sequence were aligned locally to the aggregated sequence to determine the sequence boundaries, allowing for two mismatches or indels. Bases outside the aligned boundaries were trimmed, resulting in a final aggregated sequence for each read that begins and ends with the reference sequence boundaries. In the second phase of the pipeline, all aggregated reads were globally aligned to the reference sequence using alignment reward/penalty scores (match = 1, mismatch = 0, gapOpen = −2, gapExtension = 0). These scores favored long gaps over small gaps and mismatches. Finally, all unique aggregated sequences were sorted and counted.

### 2.9. Western Blot Analysis

Western blot analysis was performed as described previously [16]. Cell lysis was performed in RIPA buffer supplemented with a protease-inhibitor cocktail (Roche, Basel, Switzerland). Protein concentrations were determined using the Pierce™ BCA Protein Assay Kit (Life Technologies, Darmstadt, Germany) following the manufacturer’s protocol. Proteins were separated in Mini-PROTEAN TGX Stain-Free Protein Gels, transferred to nitrocellulose membrane, and blocked in 5% milk/TBS-T for 1 h at room temperature. Membranes were incubated with appropriate antibodies (mouse anti-gp91*^phox^* monoclonal antibody 1:2000, anti-ß-actin antibody 1:5000) overnight at 4 °C. Following incubation with the primary antibody, membranes were incubated with corresponding secondary antibodies for 1 h at room temperature and developed using the Pierce ECL Western blotting Substrate Kit.

### 2.10. DHR123 Functional Assay

The DHR123 functional assay, adapted from Bustamante et al. [2], was employed to measure ROS production in mature macrophages and neutrophils. Initially, cells were stimulated with phorbol 12-myristate 13-acetate (PMA) at a final concentration of 0.065 µM and incubated at 37 °C for 2 h. Following this, cells were incubated with dihydrororhodamine 123 (20 µM, Sigma, Setagaya City, Tokyo) in the dark at 37 °C for 5 min, with catalase (1300 IU/mL) included to regulate the reaction. After dihydrororhodamine 123 (DHR123) incubation, the cells were washed thrice with PBS to ensure the removal of excess dye. Accutase (500 µL) was then added to detach the cells, and after a 5-min incubation at 37 °C, the reaction was stopped by adding 500 µL of FACS buffer (PBS &1% BSA). The cell suspension was transferred to a microcentrifuge tube and centrifuged at 1000× *g* for 9 min to pellet the cells. The supernatant was discarded, and the cells were resuspended in 250 µL of FACS buffer for cell counting. For the identification of macrophages, cells were incubated with 0.25 µg of APC-conjugated anti-mouse F4/80 antibody per 10^6^ cells for 30 min on ice. After the incubation, cells were washed thrice with PBS and fixed in 4% paraformaldehyde (PFA) for 20 min on ice. Following fixation, cells were washed three times with PBS and finally resuspended in 400 µL of FACS buffer. The prepared samples were stored on ice in the dark until they were subjected to immediate analysis by flow cytometry.

### 2.11. Statistical Analysis

For all experiments, data are shown as mean ± SD if not stated otherwise. Statistical analysis was performed using GraphPad Prism 9.4 (GraphPad software, Boston, MA, USA). Sample sizes and the statistical tests used are described in the figure legends. A *p* value of less than 0.05 was considered statistically significant.

## 3. Results

### 3.1. Generation of a Mouse Model Carrying a Patient-Specific Cybb Mutation

**Figure 1:**
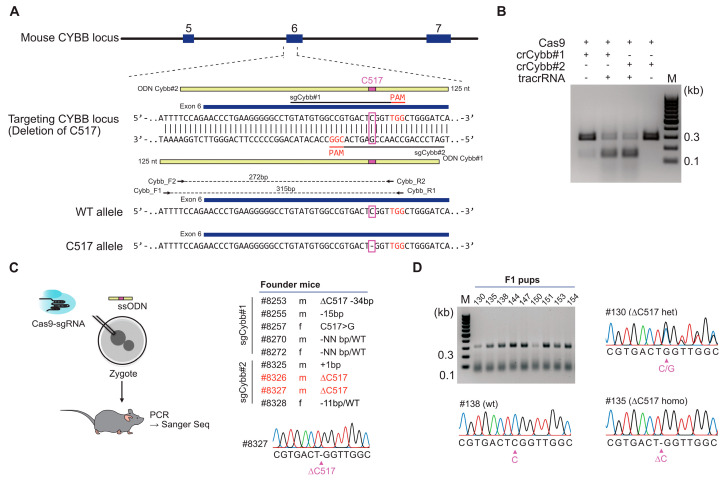
Generation of a mouse model carrying a single-nucleotide deletion (C517) in exon 6 of the *CYBB* gene using CRISPR/Cas9-mediated HDR. (**A**) Scheme illustrating the HDR strategy for targeted deletion of C517 in exon 6 of the *CYBB* gene. Two guide RNAs (sgCybb#1 and sgCybb#2) were designed to target the desired region. Single-stranded DNA templates (ODNs) with a length of 125 nucleotides were synthesized as targeting donors for each sgRNA. The ODNs were designed to be homologous to the target region, except for the absence of a C at position 517. (**B**) To assess the activity of the guide RNAs (sgCybb#1 and sgCybb#2), the target region was amplified using the primers (Cybb_F2 and Cybb_R2), as indicated in (**A**). The resulting PCR products were incubated at 37 °C with crRNAs, tracrRNA, and recombinant SpCas9. The cleavage of the PCR products was analyzed by gel electrophoresis to evaluate the efficiency of the guide RNAs in inducing DNA cleavage. (**C**) Electroporation of ODN-Cybb#2 with sgCybb#2 and Cas9 into C57BL/6 mouse zygotes. Genotyping of the resulting founder mice was performed using PCR and Sanger sequencing with the indicated primers (Cybb_F1 and Cybb_R1). The genotypes of all founder mice are summarized in the table, with those carrying the desired genotype (∆C517), indicative of successful gene editing, highlighted in red. (**D**) Genotyping of F1 progeny derived from founder mutant #8327 by PCR (using indicated primers Cybb_F1 and Cybb_R1) and Sanger sequencing.

In order to create a mouse model for X-linked chronic granulomatous disease (X-CGD), we generated an edited mouse line that carries a patient-specific Cybb mutation. The specific mutation is a deletion of a single nucleotide (C517) in exon 6 of the *CYBB* gene, which results in a frameshift beyond codon 173 and Cybb deficiency (Figure 1A). To delete this single nucleotide using CRISPR/Cas9, we selected two guide RNAs (sgCybb#1 and sgCybb#2) targeting the exon 6 region (Figure 1A). The activity of both guide RNAs was assessed using a Cas9 in vitro cleavage assay (Figure 1B). Recombinant Cas9 nuclease was incubated with the candidate crRNAs (crCybb#1 and crCybb#2), tracrRNA, and a PCR product (272 bp) amplified from the *CYBB* target DNA sequence. The resulting digestion products were analyzed by agarose gel electrophoresis to confirm the cleavage of the PCR product. The results demonstrated the activity of crCybb#1 and crCybb#2, as shown by the appearance of the predicted cleavage products (~130, ~141 bp) upon incubation with tracrRNA and Cas9 protein (Figure 1B).

For production of the mutant CYBB allele, zygotes isolated from C57BL/6 mice were electroporated with Cas9 protein, one of the validated guide RNAs (sgCybb#1 or sgCybb#2), and a modified single-stranded DNA oligonucleotide (ODN-Cybb#1 with sgCybb#1 or ODN-Cybb#2 with sgCybb#2) (Figure 1C). The DNA oligonucleotide had homology sequences flanking the targeted codon but lacked nucleotide C517 (Figure 1A,C). The induction of a double-strand break (DSB) near C517 allowed editing by homologous recombination using ODN-Cybb as a donor template, leading to the introduction of the single-nucleotide deletion into the coding region of *CYBB*. From the electroporation of C57BL/6 mouse zygotes, we obtained nine pups, which were genotyped by PCR amplification using the primer pair Cybb_F1/Cybb_R1 and sequencing of the *CYBB* exon 6 region. Among these offspring, we identified seven founder mutants carrying random indels, while two mice exhibited only the desired deletion of C517 (Figure 1C, #8326 and #8327).

Since the *CYBB* gene is located on the X chromosome, Cybb-deficient males are hemizygous for the CybbC517del allele. Since both of the founders were males, the sequence reads of the PCR product could be easily compared to the wildtype reference, confirming the deletion of C517 (Figure 1C, #8327). In the mutant allele, the single-nucleotide deletion caused a reading frameshift, leading to a stretch of 15 missense codons followed by a stop codon. 

Founder male #8327 was subsequently mated to C57BL/6 wildtype females to establish the CybbC517del mouse line. Approximately half of the offspring from this founder carried the C517del allele, therefore confirming germline transmission (Figure 1D). The CybbC517del mouse line is maintained in a heterozygous breeding scheme and housed in specified pathogen-free environment. The mutant mice are healthy and do not display a disease phenotype due to their selective immunodeficiency.

### 3.2. C517 Deletion Leads to Impaired gp91^phox^ Expression in Mature Macrophages

*CYBB* encodes the β-chain of flavocytochrome b558, also known as gp91*^phox^* or NOX2, which is a crucial component of the nicotinamide adenine dinucleotide phosphate (NADPH) oxidase complex found in phagocytes such as granulocytes, monocytes, and macrophages. While its expression is lower in other cell types like dendritic cells and B lymphocytes [2], disruptions in the *CYBB* gene commonly result in the loss of gp91*^phox^* expression due to mRNA or protein instability. This defect is often associated with frameshift and early termination of protein synthesis [9]. The CybbC517del mouse line carries a patient-specific mutation that causes a frameshift in codon 173 and leads to premature termination following 15 missense codons.

**Figure 2:**
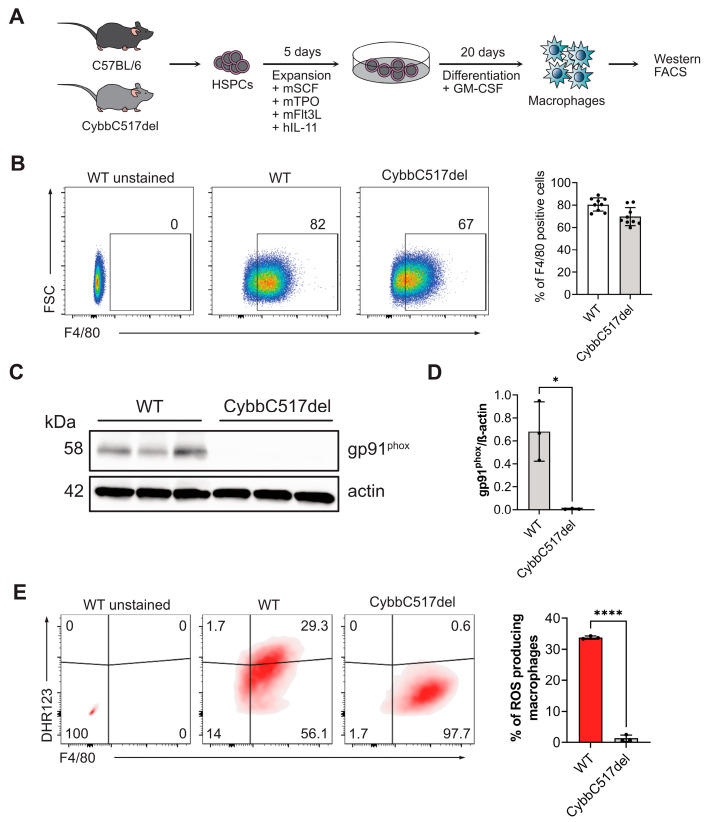
Characterization of the CybbC517del mouse model. (**A**) Schematic representation of the generation of mature macrophages from HSPCs of wild-type littermates and CybbC517del mice. After 20 days of differentiation, cells were subjected to flow cytometry and western blot analysis. (**B**) Flow-cytometry analysis of F4/80-expressing macrophages derived from wildtype littermates and mutant CybbC517del HSPCs. The gates indicate the percentages of F4/80+ cells, and each circle represents an individual mouse (12 females, 6–7 weeks) from three independent experiments (n = 3). Data are presented as means ± SD. (**C**) Immunoblot analysis of gp91*^phox^* expression in mature macrophages derived from wild-type littermates and CybbC517del mutant mice, probed with an anti-mouse gp91phox antibody and an antibody to actin (loading control). Each lane represents pooled cells from two mice of one experiment (n = 3, three independent experiments). (**D**) Quantification of densitometric analysis (ImageJ) of western blot signals showing gp91*^phox^* protein expression normalized to actin. Data are plotted as mean ± SD. Statistical significance was evaluated by a two-tailed *t* test with * *p* < 0.05. (**E**) FACS analysis (dihydrorhodamine 123 assay) of mature macrophages derived from wild-type littermates and CybbC517del. Data are plotted as mean ± SD. Statistical significance was evaluated by a two-tailed *t* test with **** *p* < 0.0001.

To validate the expected phenotype of the CybbC517del mouse line, we investigated gp91*^phox^* expression in in vitro-derived macrophages obtained from Sca1^+^ bone-marrow cells isolated from CybbC517del mutant mice (Figure 2A). In the wild-type control littermates, a specific gp91*^phox^* band at approximately 58 kDa was observed, while this band was absent in the mutant sample Figure 2C,D). The absence of the signal in the mutant sample indicates that the C517 deletion leads to impaired expression of gp91*^phox^*.

Furthermore, we investigated potential defects in the differentiation of hematopoietic stem and progenitor cells (HSPCs) resulting from the C517 deletion. To assess this outcome, we quantified and compared the population of F4/80-expressing macrophages in the mature cell pool derived from wild-type littermates and mutant HSPCs using flow cytometry. FACS analysis revealed similar quantities of mature macrophages in mutant and control (wild-type) samples (Figure 2B). As expected, these findings suggest that NADPH oxidase does not play a significant role in the development of macrophages.

### 3.3. C517 Deletion Leads to Impaired NADPH Oxidase Activity in Mature Macrophages

The phagocytic respiratory burst is a critical defense mechanism employed by the host to eliminate ingested microorganisms. It involves a series of reactions in which activated NADPH oxidase transfers electrons from cytosolic NADPH to external O_2_, resulting in the generation of superoxide anions (O_2_^−^) [8]. These superoxide anions are further metabolized into potent antimicrobial oxidants, including hydrogen peroxide (H_2_O_2_), hydroxyl anions (OH^−^), hypochlorous acid (HOCl), and nitryl chloride (NO_2_Cl) [8]. In X-CGD patients, phagocytes are unable to generate the respiratory burst due to impaired NADPH oxidase activity.

In this study, we examined the respiratory-burst activity of mature macrophages derived from HSPCs of CybbC517del mice using dihydrorhodamine 123 (DHR123) and flow cytometry. DHR123 is a non-fluorescent dye that can be oxidized to fluorescent rhodamine 123 (R123) by hydrogen peroxide, a byproduct of the respiratory burst [17].

Mature macrophages were obtained from HSPCs of CybbC517del mice, as previously described (Figure 2A). These cells were stimulated with phorbol 12-myristate 13-acetate (PMA) in the presence of catalase, and the production of H_2_O_2_ was assessed by measuring the levels of rhodamine 123 (R123).

Control macrophages derived from wild-type littermates displayed robust respiratory-burst activity, with over 30% of stimulated macrophages showing normal H_2_O_2_ production, as indicated by detectable signals of rhodamine 123 (Figure 2E). In contrast, macrophages derived from CybbC517del mutant mice failed to generate a respiratory burst, with nearly no macrophages displaying any detectable rhodamine 123 signals (Figure 2E). The absence of H_2_O_2_ production in mutant macrophages indicates impaired NADPH oxidase activity resulting from the C517 deletion. These findings confirm that the expected outcome of the patient’s mutation is consistent with our observations of gp91*^phox^* protein expression in mutant macrophages and validate the new mouse line as a model recapitulating the disease phenotype.

### 3.4. CRISPR/Cas9-Mediated Gene Correction of C517 Deletion in Mouse HSPCs

**Figure 3:**
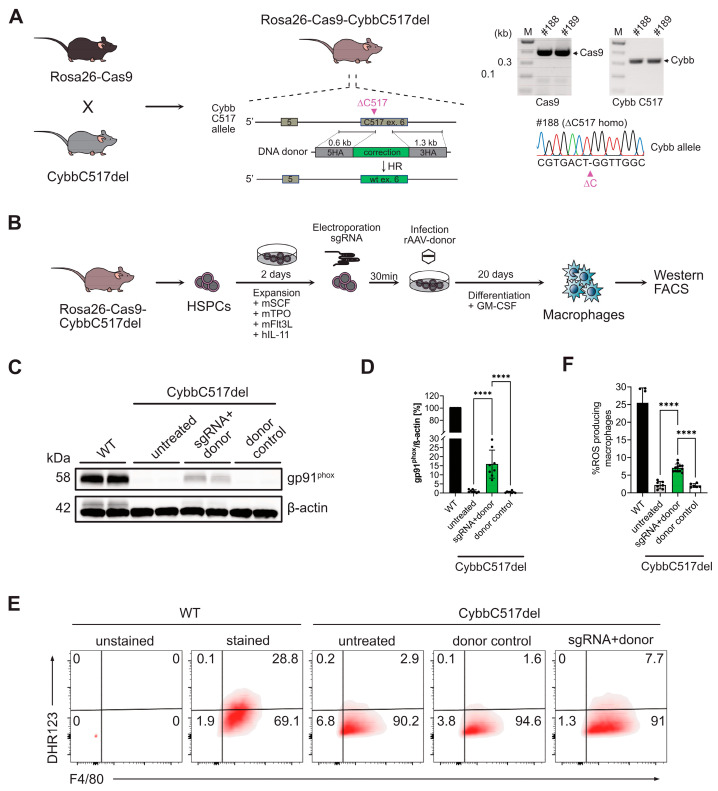
Gene correction of the C517 deletion in mouse HSPCs by CRISPR/Cas9-mediated HDR. (**A**) Generation of ROSA26-Cas9-CybbC517del mice by crossing CybbC517del mice with ROSA26-Cas9 transgenic mice. The presence of the Cas9 and C517 allele in the offspring was detected by PCR and Sanger sequencing using the indicated primers (C517: Cybb_F1 and Cybb_R1, Cas9: Cas9_F and Cas9_R). #188 and #189 represent two mice with the indicated genotypes. (**B**) Experimental scheme illustrating the gene-correction experiment in HSPCs from ROSA26-Cas9-CybbC517del mice. (**C**) Immunoblot analysis of gp91*^phox^* expression in mature granulocytes and macrophages derived from CybbC517del HSPCs after gene correction (treatment with sgCybb#3 and the AAV-6 repair template). Control samples include wild-type cells, untreated mutant cells and mutant cells treated with only the AAV-6 repair template (donor control). Each lane represents pooled cells from two mice. (**D**) Quantification of densitometric analysis (ImageJ version 1.53t) of western blot signals showing gp91*^phox^* protein expression normalized to actin. Each circle represents pooled cells of two mice (females, 6–7 weeks) from four independent experiments (n = 4). Data are presented as means ± SD. Statistical significance was evaluated by a two-tailed *t* test with **** *p* < 0.0001. (**E**) FACS analysis (dihydrorhodamine 123 assay) of mature macrophages derived from CybbC517del mutant HSPCs after gene correction (HDR: treatment with sgCybb#3 and the AAV-6 repair template). Control samples include wild-type cells, untreated mutant cells and mutant cells treated with only the AAV-6 repair template (donor control). Surface expression of F4/80 and the presence of fluorescent rhodamine 123 (R123) were evaluated. (**F**) The percentage of ROS-producing macrophages (R123+ F4/80+ cells) is shown as means ± SD, with circles representing three independent experiments (n = 3) using pooled cells from nine mice (females, 6–7 weeks).

To correct the C517del mutation in mouse HSPCs, we employed CRISPR-mediated homology-directed repair (HDR). For this purpose, we designed a repair template consisting of the wild-type coding sequence and homology arms of at least 650 bp on both sides of the targeted codon. In addition to the reintroduced missing nucleotide at position 517, the repair template also contained two silent replacements in the guide RNA and PAM sequence to prevent recurrent double-strand breaks (DSBs) in the corrected allele. To target the C517del locus, we modified the guide sequence of sgCybb#2 by extending it by one nucleotide in the 5′ direction, resulting in a gRNA termed sgCybb#3. This modification ensured that sgCybb#3 would bind with 100% complementarity to the *CYBB* mutant allele. To test our strategy, we conducted the gene-editing experiment using HSPCs from CybbC517del mice with constitutive Cas9 expression. To achieve this, we crossed CybbC517del mice with ROSA26-Cas9 transgenic mice, which express Cas9 ubiquitously from the ROSA26 locus (Figure 3A).

To assess HDR efficiency, we isolated Sca1^+^ HSPCs from ROSA26-Cas9- CybbC517del mice, activated them with mouse SCF, TPO, Flt3L, and human IL-11 for 2 days, and then performed electroporation with Cas9 and sgCybb#3. The pretreated HSPCs were subsequently infected with recombinant AAV-6 viral vectors carrying the repair templates (AAV-Cybb) at a multiplicity of infection (MOI) of 1,000,000 viral genome copies (GCs) per cell. The targeted HSPCs were then differentiated into macrophages in the presence of GM-CSF for the next 20 days (Figure 3B). After targeting and differentiation, we quantitatively evaluated the HDR efficiency of the edited mature macrophages by analyzing the recovery of gp91*^phox^* using western blot analysis (Figure 3C). In the experimental groups receiving both sgRNA and the AAV-6 repair template (HDR), we observed a 16% recovery of gp91*^phox^* expression. In contrast, untreated cells and cells receiving only the AAV-6 repair template (donor control) did not exhibit expression of the gp91*^phox^* protein (Figure 3C,D). Furthermore, cells treated with both sgRNA and the AAV-6 repair template (HDR) showed improved respiratory-burst activity, with 7% of stimulated macrophages displaying normal H_2_O_2_ production, compared to 2% in untreated cells and donor control-treated cells (Figure 3E,F).

**Figure 4:**
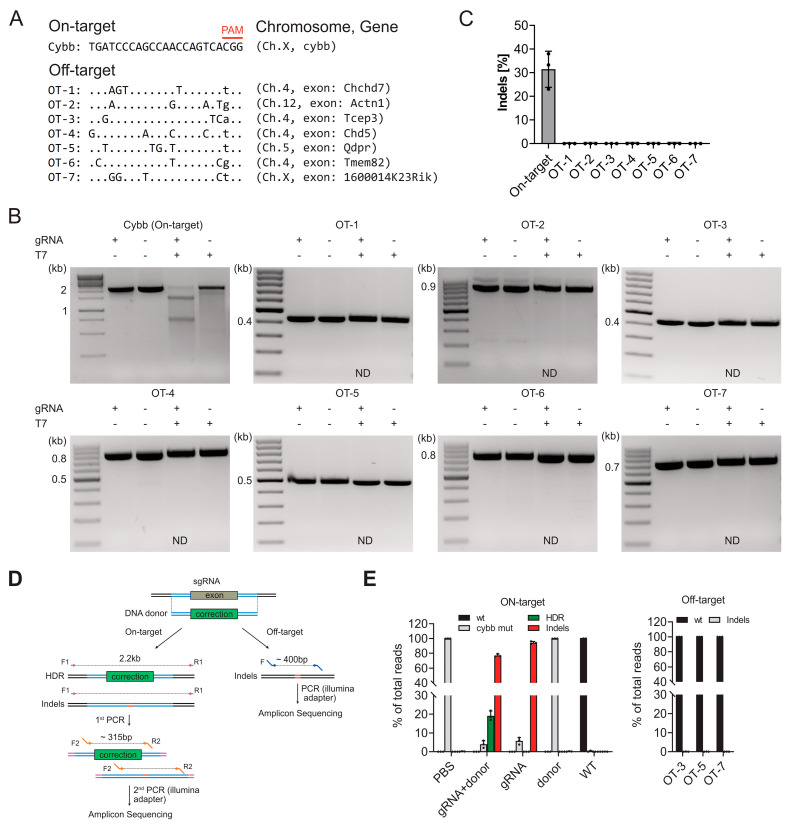
Off-target analysis in mutant HSPCs after gene correction. (**A**) On-target and the seven most prominent off-target sites found in exonic regions, as predicted by CRISPOR. (**B**) Estimation of indel frequencies at the on-target and off-target sites using the T7 endonuclease I (T7EI) assay (ND, not determined). (**C**) Densitometric analysis (ImageJ version 1.53t) of T7EI results showing indel frequency in on- and off-target sites. Data are presented as means ± SD from three independent experiments. (**D**) Schematic representation illustrating the deep-sequencing strategy for editing events at on- and off-target sites in mature macrophages derived from CybbC517del HSPCs after gene correction. (**E**) Amplicon sequencing of selected on-target and off-target sites in wildtype, untreated, and treated cells. Data are presented as means ± SD, with circles representing three independent experiments (n = 3) using pooled cells from nine mice (females, 7–8 weeks).

To genetically confirm the correction events, we amplified the target region from genomic DNA of wild-type, untreated, and treated cells and quantified the editing events through amplicon sequencing (Figure 4D). In cells treated with both sgRNA and the AAV-6 repair template, we observed a 19% HDR correction of the pathogenic mutation (Figure 4E). In comparison, untreated cells and cells treated with sgRNA alone did not show any correction (Figure 4E). The frequencies of indels were high, reaching 77% in cells treated with sgRNA alone and 94% in cells treated with sgRNA and the repair template (Figure 4E). These results collectively demonstrate that our HDR strategy successfully corrects the patient-derived C517 deletion in primary HSPCs and partially rescues the molecular and phenotypic consequences of the mutation.

### 3.5. Off-Target Analysis in Edited HSPCs

To assess the safety and specificity of our gene-editing approach, we performed an off-target analysis in edited HSPCs. Off-target effects refer to unintended modifications occurring at genomic sites other than the intended target locus. Using the online tool CRISPOR (https://crispor.tefor.net/ (accessed on 14 May 2021)), we identified a list of 7 potential off-target sites that exhibit moderate sequence similarity to the target site, with 3–4 mismatches (Figure 4A). Notably, all of these off-target sites were located within exonic regions of genes. The presence of off-target sites in exonic regions raises specific concerns, as modifications within these regions have a higher likelihood of affecting gene function and potentially leading to unintended consequences. Therefore, our primary focus was directed towards evaluating the potential off-target effects within exonic sequences.

To assess the presence of off-target effects, we amplified all seven off-target sites in sgCybb#3 in the targeted HSPCs. Initially, we estimated the indel frequencies at both the on-target and off-target sites using T7 endonuclease I (T7EI) assay (Figure 4B). The results of this assay revealed an indel frequency of 31% at the on-target site, while none of the off-target sites led to a detectable cleavage product, suggesting that non-homologous end joining (NHEJ) was not evident at these off-target sites (Figure 4C). To validate these data and obtain a more comprehensive analysis, we performed amplicon sequencing of three selected off-target and on-target sites in wild type, untreated, and treated cells (Figure 4D). Consistent with the results of the T7EI assay, the amplicon-sequencing data demonstrated no detectable off-target mutations or indels at the predicted off-target sites in the edited HSPCs (Figure 4E). These data suggest that our gene-editing approach exhibited high specificity and demonstrated low off-target activity. Notably, the indel frequency at the on-target site observed in the amplicon sequencing data was approximately three-fold higher in the edited samples compared to the indel frequency obtained from the T7EI assay (Figure 4E). This difference in indel frequencies can be attributed to the different sensitivities and methodologies employed by the two assays. This highlights the importance of employing multiple complementary approaches to thoroughly evaluate potential off-target effects and ensure the accuracy of the findings.

## 4. Discussion

Our study presents the first mouse model (CybbC517del) carrying a patient-derived mutation for X-linked Chronic Granulomatous Disease (X-CGD). The creation of this transgenic mouse model using CRISPR/Cas9 to introduce a single-nucleotide deletion (C517) in the *CYBB* gene provides a valuable tool for studying X-CGD.

Notably, the model demonstrates the clinical relevance of the mutation, as evidenced by impaired gp91*^phox^* expression and NADPH oxidase activity in mature granulocytes and macrophages, mirroring the pathophysiology observed in classical human X-CGD patients. Our findings show that, despite the impaired NADPH oxidase activity, the development of macrophages is unaffected in CybbC517del mice. This observation is consistent with the current understanding that NADPH oxidase primarily functions in microbial killing rather than in cell development within the hematopoietic lineage. The absence of a respiratory burst in macrophages derived from CybbC517del mice further substantiates the model’s validity in replicating the human disease phenotype.

While Pollock et al. [18] previously described a gp91*^phox^* (*CYBB*)-deficient knockout mouse model, our CybbC517del model represents a significant improvement in modeling X-linked Chronic Granulomatous Disease (X-CGD). The use of a patient-derived mutation allows for a more accurate representation of the disease pathology. Moreover, the CybbC517del model provides a unique opportunity to study the specific impacts of individual mutations within the *CYBB* gene, offering insights into mutation-specific disease mechanisms and responses to therapy.

Another important aspect of this study is the use of CybbC517del mice as a novel platform for the evaluation of potential gene-repair strategies using CRISPR/Cas9. We employed homology-directed repair (HDR) with a tailored repair template for the correction of the C517 deletion. Our approach achieved a significant correction rate and partial restoration of gp91*^phox^* expression and NADPH oxidase activity. These results underscore the potential of CRISPR/Cas9-based therapies for the treatment of genetic disorders like X-CGD. However, the observed partial recovery of function highlights the need for further optimization of our gene-editing strategies to achieve complete therapeutic efficacy.

Our off-target analysis provides important insights into the safety and specificity of the gene-repair approach. The lack of detectable off-target effects at predicted sites reaffirms the precision of our strategy. However, we observed high indel frequencies at the on-target sites, a phenomenon that may be amplified by our use of mouse HSPCs with constitutive Cas9 expression. Future experiments using transient Cas9 expression mediated by RNP complexes might be necessary to provide a more comprehensive evaluation of the efficiency and safety of our editing approach. Nonetheless, the observed high indel frequencies highlight a limitation of HDR using traditional Cas9 nucleases, underscoring the need for alternative strategies to minimize non-homologous end joining (NHEJ)-mediated on-target mutations. The Spacer Nick approach described by Tran et al. [15] represents a promising strategy for enhancing the precision and effectiveness of gene editing in such contexts. This approach is based on the placement of distant single-strand break in the target gene using Cas9 nickase to stimulate recombination but lead to a strongly reduced indel profile at the on-target and off-target sites.

While Ravin et al. [10,19] have previously demonstrated the feasibility of correcting a Cybb mutation (exon 7) in human HSPCs from X-CGD patients, our approach extends these findings by using a mouse model. In addition, the CybbC517del model allows us to overcome one limitation faced when using human HSPCs, that is the scarcity of patient-derived HSPCs.

A significant challenge in ex vivo gene-repair strategies such as the ones employed in our study and by Ravin et al. [10,19] is the difficulty of acquiring a sufficient number of viable, corrected cells for successful transplantation after gene editing. In this context, in vivo gene editing, which involves directly correcting genes within the living organism, emerges as a potential solution to bypass these limitations. However, this approach is complex and the translation of in vivo gene editing from experimental models to clinical application requires extensive evaluation, particularly regarding its safety and efficacy. In this regard, our model could offer a controlled environment in which to test the feasibility, safety, and outcomes of in vivo editing approaches.

Our results suggest that the CybbC517del mouse model can serve as a platform not only for further investigation of X-CGD pathophysiology, but also for the refinement and evaluation of novel gene-therapy strategies. Future studies using this model could explore the long-term efficacy and safety of corrected HSPCs in vivo, assess the immune response following transplantation, and evaluate the potential of in vivo gene-editing approaches. Additionally, this model could be instrumental in investigating the broader implications of NADPH oxidase deficiency in immune function and microbial defense, potentially uncovering new therapeutic targets.

In conclusion, our study highlights the power of CRISPR/Cas9-mediated genome engineering in creating accurate disease models and developing gene-therapy strategies. Such models will be pivotal in bridging the gap between laboratory research and clinical applications, bringing us closer to effective treatments for genetic disorders like X-CGD.

## Figures and Tables

**Table 1:** List PCR primers. Sequence (5′–3′).

Amplicon Sequencing Off-Target Primers	
Chchd7_for	ACACTCTTTCCCTACACGACGCTCTTCCGATCTCGATATATTGAGATAAATGAAGCCC
Chchd7_rev	GACTGGAGTTCAGACGTGTGCTCTTCCGATCTCACAAACAAAAGTCAAGTTACTGC
Tceb3_for	ACACTCTTTCCCTACACGACGCTCTTCCGATCTAGCATAGCAATGCACAGTAAGG
Tceb3_rev	GACTGGAGTTCAGACGTGTGCTCTTCCGATCTGCTGTGGAACTTGCTTTTGTT
Qdpr_for	ACACTCTTTCCCTACACGACGCTCTTCCGATCTTTCGAGAGGGCTCTCCCAGG
Qdpr_rev	GACTGGAGTTCAGACGTGTGCTCTTCCGATCTCCACCCTTCACACCCAAGGC
Actn1_for	ACACTCTTTCCCTACACGACGCTCTTCCGATCTTTTGTGGGCAGGGAGGCTTG
Chd5_for	ACACTCTTTCCCTACACGACGCTCTTCCGATCTAGTGGCTCTGTGGGAGGAGG
Chd5_rev	GACTGGAGTTCAGACGTGTGCTCTTCCGATCTTGTGCAGGACAGCATTGGCA
Tmem82_for	ACACTCTTTCCCTACACGACGCTCTTCCGATCTTGGTGCATGGCCACACCTTC
Tmem82_rev	GACTGGAGTTCAGACGTGTGCTCTTCCGATCTCGATTCTGGACCCCGCTCAC
K23Rik_for	ACACTCTTTCCCTACACGACGCTCTTCCGATCTGCTCCCTGACCCTTGGGAGA
K23Rik_rev	GACTGGAGTTCAGACGTGTGCTCTTCCGATCTTGCTGTGGCAGAAAGTGGCA
Amplicon sequencing on-target primers	
Cybb-Amplicon-for	ACACTCTTTCCCTACACGACGCTCTTCCGATCTAGCAGTTCTGAGCCTGTGT
Cybb-Amplicon-rev	GACTGGAGTTCAGACGTGTGCTCTTCCGATCTGCAAGGCCGATGAAGAAGAT
Genotyping PCR primers and T7EI primers for target site	
Cybb-geno-for	ATAACCGATGAGTGCAGGCT
Cybb-geno-rev	GACATGGCTTGGGAAACACA
Cybb-for1	AAATCTCAACACCAGATCTGAGAG
Cybb-rev1	CTCAGCTCCATGGATGGCAAGG
Cas9-for	GGCATCCTGCAGACAGTGAAGGTGG
Cas9-rev	CGGTTCTTGTCGCTTCTGGTCAGCA
T7EI primers for off-target sites	
Actn1-T7-for	GGCCAGCCACACTTTTGAAG
Actn1-T7-rev	ACCAAGCCGAGTACTGCATC
Chd5-T7-for	TCACCCCTGAGGAGAGTCAG
Chd5-T7-rev	CCCAGGTCAGAAAGGCAGAG
Tmem82-T7-for	GTCCTCAAGACCAACCCAGG
Tmem82-T7-rev	CACACGGTTTGCCTGTGAAG
1600014K23Rik-T7-for	CACCTGGTTCAATCCCCTCC
1600014K23Rik-T7-rev	CGCCCATCTCCTTGAGGATC
Chchd7-T7-for	CGATATATTGAGATAAATGAAGCCC
Chchd7-T7-rev	CACAAACAAAAGTCAAGTTACTGC
Tceb3-T7-for	AGCATAGCAATGCACAGTAAGG
Tceb3-T7-rev	GCTGTGGAACTTGCTTTTGTT
Qdpr-T7-for	CCATATACCTCCCTGGACCC
Qdpr-T7-rev	TTCTTTGGGGACCTGTGTATATG
AAV titration qPCR primers	
ITR-for	CGGCCTCAGTGAGCGA
ITR-rev	GGAACCCCTAGTGATGGAGTT
Taqman probe	FAM-CACTCCCTCTCTGCGCGCTCG-BHQ

red: Illumina adapter.

## Data Availability

The raw data supporting the conclusions of this article will be made available by the authors on request.
